# Diffusion tensor imaging and white matter abnormalities in patients with disorders of consciousness

**DOI:** 10.3389/fnhum.2014.01028

**Published:** 2015-01-06

**Authors:** Carlo Cavaliere, Marco Aiello, Carol Di Perri, Davinia Fernandez-Espejo, Adrian M. Owen, Andrea Soddu

**Affiliations:** ^1^Coma Science Group, Cyclotron Research Center and Neurology Department, University and University Hospital of LiegeLiege, Belgium; ^2^IRCCS SDN, Istituto Ricerca Diagnostica NucleareNaples, Italy; ^3^Neuroradiology Department, National Neurological Institute C. MondinoPavia, Italy; ^4^Psychology Department, Brain and Mind Institute, University of Western OntarioLondon ON, Canada; ^5^Physics and Astronomy Department, Brain and Mind Institute, University of Western OntarioLondon ON, Canada

**Keywords:** coma, brain, diffusion tensor imaging, consciousness, tractography

## Abstract

Progress in neuroimaging has yielded new powerful tools which, potentially, can be applied to clinical populations, improve the diagnosis of neurological disorders and predict outcome. At present, the diagnosis of consciousness disorders is limited to subjective assessment and objective measurements of behavior, with an emerging role for neuroimaging techniques. In this review we focus on white matter alterations measured using Diffusion Tensor Imaging on patients with consciousness disorders, examining the most common diffusion imaging acquisition protocols and considering the main issues related to diffusion imaging analyses. We conclude by considering some of the remaining challenges to overcome, the existing knowledge gaps and the potential role of neuroimaging in understanding the pathogenesis and clinical features of disorders of consciousness.

## Introduction

The need to explain clinical and behavioral abnormalities in different neurological conditions in the absence of definite brain lesions to conventional imaging techniques has boosted the use of “non-conventional” magnetic resonance imaging (MRI) techniques to investigate apparently normal brain tissue (Molino et al., 2014). Diffusion tensor imaging (DTI) is a non-invasive imaging technique implemented in MRI (Basser et al., 1994), which can detect WM alterations that are not visible using conventional imaging techniques such as computed tomography (CT) and MRI (Arfanakis et al., 2002; Hulkower et al., 2013). DTI has been used in both research and clinical settings and has provided valuable biomarkers for tissue injury severity and outcome predictors (Tshibanda et al., 2009). DTI has also found application in several studies related to brain maturation, detecting age-related degeneration in healthy subjects (Sullivan et al., 2001) and patients with cerebral ischemia (Pierpaoli et al., 2001) or traumatic brain injury (TBI) with related diffuse axonal injury (Yeh et al., 2012). Moreover, despite significant variation in sample characteristics, experimental setup, technical aspects of imaging and analysis approaches, DTI's valuable contribution to brain pathologies, including the large spectrum of disorders of consciousness (DOC), is now recognized (Hulkower et al., 2013). Whilst progress has been made describing DOC from a clinical perspective (Bruno et al., 2012), the present paper focuses on examining DOC patients from a point of view of recent developments deriving from studies of “normal appearing” WM (Fernández-Espejo et al., 2010). We will review the main studies on white matter alterations measured using DTI techniques in patients with DOC and examine the most common DTI protocols focusing on the main issues related to DTI analyses. In our opinion, new insights obtained by studying white matter alterations using DTI (Tshibanda et al., 2009; Zappalà et al., 2012) could better our understanding of DOC, the diagnosis of these conditions and possibly prognosis and treatment (Huisman et al., 2004; Perlbarg et al., 2009; Newcombe et al., 2011).

## DTI protocols and settings

The physical principle behind DTI is molecular diffusion: any molecule in a fluid is randomly displaced by means of thermal energy (Brownian motion). The first magnetic resonance sequence able to measure diffusion coefficient in structures was developed in the early 80's (Wesbey et al., 1984), paving the way for a new, well-established imaging contrast for *in vivo* quantification of molecular diffusivity. Although a variety of sequences are now used to acquire diffusion weighted (DW) images, all DW sequences include two equal and opposing motion-probing gradients (Nucifor et al., 2007). The acquired signal from a voxel will exponentially decay as function of a parameter b which determines the strength and duration of the diffusion gradients and essentially represents the measurement's sensitivity to water diffusion. The fact that the different diffusion contrasts depended upon the relative angle between the diffusion gradient and the direction of the neuronal fiber tracts became evident in the early 90's (Moseley et al., 1990). Such evidence rapidly led to the concept of the diffusion tensor, a 3 × 3 symmetrical matrix characterized by 6 independent values which describes how the driving force generating water molecule diffusion in different tissues results in a complex pattern of molecule movement. The diffusion tensor permits us to extract scalar diffusion properties such as mean diffusivity (MD) or fractional anisotropy (FA). Moreover, the principal directions of the diffusion tensor can be used to infer the dominant direction of the neural fiber tracts (Filler, 2009). More specifically, DTI reveals two specific characteristics: diffusion anisotropy and the directional distribution of water diffusivity. When water diffusion in a tissue is more or less the same in all directions, the diffusion is considered isotropic. Conversely, when water diffusion is restricted to a specific direction, the diffusion is considered to be anisotropic.

In order to achieve meaningful information for the calculation of the diffusion tensor, at least 6 diffusion-weighted (DW) images for each section are needed, however, depending on the focus of the different DTI applications, more directions (up to 128) may be needed (Ni et al., 2006). In order to minimize acquisition time and, subsequently, motion artifacts from the patient's movement, the most common technique is spin-echo planar imaging. This is a fast imaging technique which has the advantage of acquiring a comprehensive set of DW brain volumes in a relatively short time. Furthermore, DTI can benefit from recent advances in actively shielded 3.0 T magnets, improving its performance (Okada et al., 2006) in terms of higher temporal and spatial resolution and signal to noise ratio. A well-known disadvantage of high field echo-planar imaging is higher sensitivity to susceptibility artifacts, which leads to geometric distortions and signal dropouts near tissue/air and tissue/bone interfaces (Le Bihan et al., 2006). Starting from each slice of a DW volume, it is possible to carry out quantitative information about brain diffusivity features. Regarding scalar DW imaging, the apparent diffusion coefficient (ADC), which is currently that most commonly used for clinical applications, is calculated by collecting at least 2 different *b*-values. Since the measured properties are strictly dependent on *b*-values and inspected diffusion directions, ADC can also be carried out for each direction of DTI. When using DTI it is common procedure to apply a useful technique of standard linear algebra (diagonalization) for each voxel to determine the principal value (eigenvalues) of the diffusion tensor. This procedure also provides a set of 3 independent vectors which represent the principal directions of the diffusion tensor (eigenvectors) associated with each eigenvalue. The diffusion tensor is, therefore, usually represented and visualized by an ellipsoid with the eigenvectors defining the directions of the principle axes and the ellipsoidal radii defined by the eigenvalues.

After diagonalization it is possible to obtain some meaningful voxel-wise parameters. The simplest way to summarize the diffusivity property of a voxel is to average the ADC along all directions, or, more robustly, to calculate the mean eigenvalue, also named as mean diffusivity (MD) (Basser and Pierpaoli, 1996; Cercignani et al., 2001). Fractional Anisotropy (FA) is carried out via the analysis of the differences between the three eigenvalues, quantifying the degree of anisotropy, resulting on FA maps with strongly enhanced regions with high directional diffusivity (WM).

## DTI analyses

Tractography or fiber-tracking commonly refers to the process of reconstructing the fiber bundle trajectories derived from the voxel-wise DT information. Reconstruction of fiber bundle trajectories from DTI data assumes that, from a given voxel in the brain, a pathway or fiber tract can be defined by looking forward and backward along the direction of the principal eigenvector (Jones et al., 2000). Even though this is the most widely used method, the quality of the data is of upmost importance; acquisition noise seriously affects the eigenvectors extraction procedure (Le Bihan et al., 2006). However, a more important shortcoming of DTI is its inability to correctly deal with voxels containing several populations of fibers that do not necessarily have the same orientation but, instead, are arranged in more complex configurations such as crossing, kissing and branching of fiber bundles (Alexander et al., 2002; Wedeen et al., 2012). For instance, if two bundles cross at 90°, the principal eigenvector of such a voxel does not have a clear relation to the underlying structure orientation and may be the source of abrupt (non-anatomical) interruptions of fiber tracts inside WM or erroneous connections between adjacent fibers (Le Bihan et al., 2006). To address this shortcoming, advanced emerging DW techniques such as high-angular-resolution diffusion imaging with high *b*-values (HARDI) (Alexander et al., 2002; Tuch et al., 2002; Hess et al., 2006) and probabilistic tractography (Behrens et al., 2003) have been proposed. By means of a large number of encoding directions, the diffusion distribution is quantified without “a priori” model information. A probability distribution function is directly derived from the acquired data and transformed into the orientation distribution function (ODF) whose local maxima corresponds to the dominant diffusion directions. Fiber orientation is obtained using either the diffusion orientation density function (dODF) or the fiber orientation density function (fODF) (Jones et al., 2013). Examples of dODF-based techniques are the well known Q-ball imaging (QBI) approach (Tuch et al., 2002; Tuch, 2004; Descoteaux et al., 2007), which estimates ODF using Funk-Radon Transform (FRT) on diffusion data represented with spherical harmonics (SH) and diffusion-spectrum imaging (DSI) (Lin et al., 2003). A valuable example of an fODF-based technique is spherical deconvolution (SD) (Tournier et al., 2004; Dell'Acqua et al., 2013).

Once the acquisition and processing parameter procedures are addressed for a specific purpose, data analysis can be performed either on a regional or a whole-brain scale. The choice of analysis method depends highly on various factors, principally the acquisition protocol and the available data sample. In order to detect abnormalities in the neuronal tracts associated with specific symptoms or status, the direct approach involves looking at the quantitative parameters (FA, MD, ADC) associated with the voxels of the corresponding fibers. These parameters are then compared to those extracted from a normal control population to reveal any significant group differences. This approach has also been demonstrated to be suitable for single subject analysis (Grossi et al., 2012; Molino et al., 2014). Probabilistic methods, instead, produce maps of “connectivity,” which represent the probability of a specific voxel to be connected to a reference seed region (Yeh et al., 2012).

**Figure 1:**
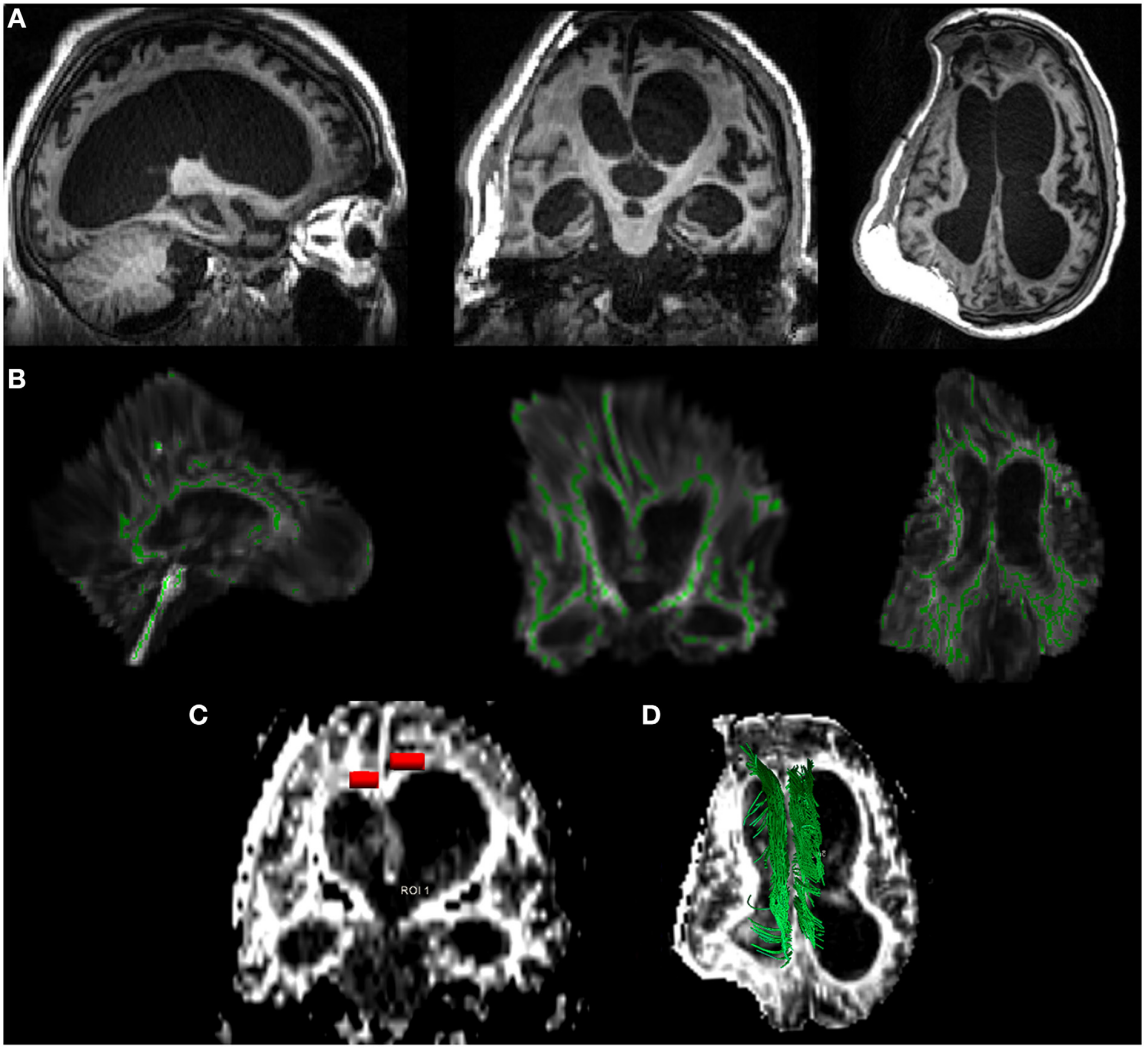
**DTI analysis in a case of 26-year-old patient, 15 months post-traumatic brain injury**. In **(A)**, brain T1 images along the three orthogonal axes, showing skull/brain deformation of the right hemisphere, severe hydrocephalus, and frontal bilateral lesions. In **(B)**, the tract based spatial statistics—TBSS—approach: the TBI brain is automatically normalized to the Montreal Neurologic Institute atlas (MNI) with the white matter skeleton (green) superimposed. The limits of this approach in severe injured patients are clear (e.g., brain distortion and white matter/skeleton alterations). In **(C)**, a typical ROI-based approach (red) applied to both the cingulum regions: a hand-made ROI is manually selected by an expert radiologist (CC) on the cinguli to extract DTI parameters within the selection. In **(D)** a deterministic approach to the cinguli (green): the tracts are manually identified and reconstructed to evaluate the entire tract for further extraction of DTI parameters overall the examined fascicle.

The region of interest (ROI)-based approach is highly related to the choice of the fibers' tracts which are usually identified as fibers crossing a manually defined region. Such a technique requires a highly experienced operator and is not, therefore, easily reproducible. To circumvent this problem, a whole brain approach of tract-based spatial statistics (TBSS) (Smith et al., 2006) has been introduced to identify WM abnormalities via voxel-wise statistical analysis of the different groups' quantitative parameters. TBSS aims to improve the sensitivity, objectivity and interpretability of the analysis of multi-subject diffusion imaging studies. With respect to ROI-based analysis, TBSS is less suitable for single-subject analysis and, due to unavoidable spatial normalization, it is difficult to apply when there are severe alterations of MR structural data, as they could compromise registration to a morphological template (Figure 1). These issues are also valid for other automatic whole brain approaches, like as TRACULA (TRActs Constrained by UnderLying Anatomy) (Yendiki et al., 2011) which uses probabilistic tractography on normalized datasets to automatically reconstruct a set of major WM pathways. Moreover, the applications and limitations of more recent techniques, like probabilistic tractography, remain controversial, considered that the probability of individual maps do not represent a direct measure of the anatomical probability of the tract (Dell'Acqua and Catani, 2012).

## State of the art in DOC patients

DTI is a relatively new MRI technique for the characterization of comatose patients (Hulkower et al., 2013) if we consider that the earliest research article reporting DTI applied to TBI was published in 2002 (Tshibanda et al., 2009). Up to now, numerous articles have been published on this topic, focusing mainly on traumatic etiology. Other authors have tried to differentiate by DTI the different conscious states [e.g., vegetative state (VS) vs. minimally conscious state (MCS)] using DTI on a combined sample of TBI and non-TBI patients (Fernández-Espejo et al., 2011) or to correlate DTI findings with injury severity and clinical outcome (Perlbarg et al., 2009; Fernández-Espejo et al., 2010, 2012; Bruno et al., 2011; Newcombe et al., 2011). The “a priori ROI” approach was the preferred choice for data analysis.

**Figure 2:**
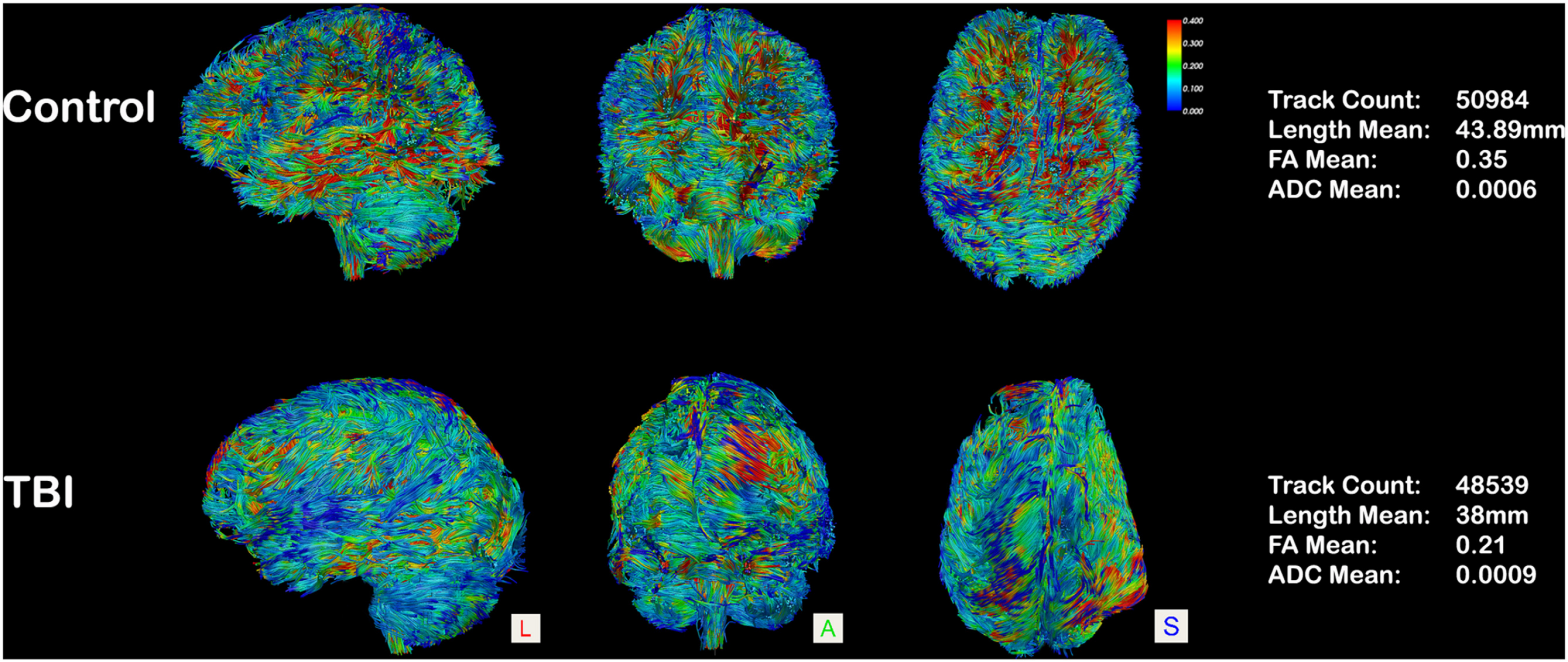
**Whole-brain tractography in an aged-matched control and in a case of 26-year-old patient, 15 months post-traumatic brain injury**. Tracts are represented in a scalar mode (0.4–0): regions with higher FA values are colored in red (0.4) and the ones with lower FA (0) in blue. A diffuse reduction of FA values is evident in the TBI patient (blue prevalence), and quantified on the right column.

Regarding the anoxic/hypoxic injured (AHI) patients, very few studies have tried to characterize microstructural white matter alterations detected by DTI. Recently, a case report on a 76-year-old man with vegetative state (VS) syndrome examined with DTI at day 41, 75, 173, and 284 after cardiac arrest, reported an early FA reduction in eleven regions of interest within the cerebral WM that preceded macroscopical MR and post-mortem neuropathological findings (Gerdes et al., 2014). Another study performed on 49 cardiac arrest patients (van der Eerden et al., 2014) demonstrated a significant reduction of axial and radial (along and across the fiber) diffusivity in 19 predefined cerebral WM areas. Other authors (Wu et al., 2011; Luyt et al., 2014), using the same ROIs approach, investigated FA and ADC alterations respectively in 57 and 49 comatose survivors of cardiac arrest. The authors concluded that FA values are highly dynamic following injury, and its alteration can predict an unfavorable outcome with sensitivity and specificity of about 94 and 100%, respectively (Luyt et al., 2014). A further study involving five VS anoxic patients has recently reported, at a minimum of 3 months post-injury, a diffuse reduction of FA values in the preselected supratentorial area (such as thalamus and corpus callosum) without significant differences in the infratentorial compartment (such as pons and midbrain areas) (Newcombe et al., 2010). These alterations have been shown to correlate with the Revised Coma Recovery Scale (CRS-R) score and functional magnetic resonance imaging (fMRI) activation following an auditory task (Newcombe et al., 2010). As for TBI patients, the first study involving DTI in Diffuse Axonal Injury was published in 2002 (Arfanakis et al., 2002). In particular, the authors performed DTI on 10 control subjects and on 5 patients with mild traumatic brain injuries examined within 24 h of injury. In the case of all patients, reduced FA values were reported in regions without macroscopic lesions, principally the internal capsule and corpus callosum. These findings are supported by other more recent studies (Rutgers et al., 2008; Wang et al., 2008) which compared controls to TBI patients (Figure 2). They reported a significant reduction of FA in both the callosal genu and splenium regions, and a reduction in the number of splenium-reconstructed fibers for severe TBI (Rutgers et al., 2008). Moreover, the alterations in the genu and splenium of the corpus callosum correlated significantly with the Revised Glasgow Outcome Scale and the outcome of the patients in question. Other studies on severe TBI patients (Kraus et al., 2007; Xu et al., 2007; Newcombe et al., 2011; Galanaud et al., 2012; Edlow et al., 2013) showed altered diffusion parameters, and mainly FA, in many regions of interest (including anterior and posterior corona radiata, cortico-spinal tracts, cingulum fiber bundles, external capsule, forceps minor and major, genu, body and splenium of the corpus callosum, inferior fronto-occipital fasciculus, superior longitudinal fasciculus and sagittal stratum). Again, these alterations were negatively correlated with all cognitive domains (Kraus et al., 2007) and clinical outcome (Newcombe et al., 2011). Perlbarg et al. (2009), in patients with severe TBI, reported significant differences of FA but not ADC values between favorable and unfavorable 1-year outcome groups in four FA tracks: inferior longitudinal fasciculus, posterior limb of the internal capsule, cerebral peduncle, and posterior corpus callosum. Finally, another study (Bruno et al., 2011) has highlighted the complementarities of DTI with other neuroimaging techniques, correlating DTI findings with two very different outcomes in otherwise similar patients and suggesting, hence, the importance of multimodal neuroimaging in the assessment of DOC patients.

Other interesting experimental setups have been prompted to elucidate white matter alterations in TBI patients. A few studies (Edlow et al., 2013; McNab et al., 2013) adopted both the deterministic and probabilistic tractography analysis approaches to ascertain the role of the ascending arousal reticular system (ARAS) in a postmortem study of a traumatic coma patient. In particular, a complete disruption of WM pathways connecting brainstem arousal nuclei to the basal forebrain and thalamic nuclei, with partial preservation of the thalami-cortical pathways was reported (Edlow et al., 2013). A very recent study (Jang et al., 2014) using probabilistic tractography on 14 AHI patients and 10 control subjects has confirmed a decrease in FA values and tract volume in the lower portion of the ARAS without significant differences in MD value. Another study (Fernández-Espejo et al., 2012) used probabilistic tractography on patients with different DOC states (VS, MCS, exit-MCS) and controls to explain differences in the structural connectivity of the default mode network that correlate with clinical diagnosis and CRS-R. The prospective setup is rarely performed on such patients (Arfanakis et al., 2002; Dinkel et al., 2014). A follow-up study of up to 5 years post-injury, has recently been published regarding 13 severe TBI patients (Dinkel et al., 2014). The authors described DTI alterations in 20 predefined WM regions and the dynamic evolution of these parameters with the clinical outcome of patients. Another study has investigated DTI alterations to differentiate between MCS and VS patients (Fernández-Espejo et al., 2011), identifying different subcortical WM and thalamic regional alterations. The authors concluded that DTI can predict the appropriate diagnostic categories (MCS vs. VS) with an accuracy of 95%. Finally, a handful of studies have compared DTI findings in patients with coma due to different etiologies. Newcombe et al. (Newcombe et al., 2010) identified supra/infratentorial differences between TBI (*n* = 7) and AHI (*n* = 5), reporting distinctive infratentorial involvement in cases of traumatic etiology. van der Eerden et al. (2014), by combining different DTI parameters in 49 cardiac arrest and 40 TBI patients at 5–57 days after insult, recently reported a predominance of cerebral hemisphere axonal injury in cardiac arrest cases and central myelin injury in TBI.

## Limits and considerations

DTI has been used extensively to identify brain abnormalities in TBI and correlate them with other clinical features related to the disorder (Hulkower et al., 2013). However, DTI studies of AHI are scarce (van der Eerden et al., 2014), thus highlighting an area which offers great opportunity to understand the anatomical and functional brain basis of the pathogenesis and symptoms of AHI, and any meaningful connectivity dysfunction or micro-structural changes that overlap or are distinct from patients with TBI. The main advantage of using DTI on DOC patients is that one can evaluate white matter micro-structural integrity under sedation (water diffusion properties are not theoretically affected by sedatives or hypnotics). This feature is a key factor that can importantly restrict artifacts in DTI data due to the involuntary movements common to DOC patients' acquisitions, the likes of which severely hamper other advanced MRI techniques such as functional magnetic resonance imaging (fMRI), and sometimes even the assessment of clinical scores (Tshibanda et al., 2009). Moreover, the possibility to acquire good quality DTI data in DOC patients labels DTI a technique with considerable alternative prognostic power, as low initial GCS scores are of limited value when predicting outcome (Huisman et al., 2004). As for fMRI analyses, DTI techniques suffer problems related to brain normalization, which is essential to perform group analyses when local properties are the subject of interest (Soddu et al., 2011). In fact, patients with chronic DOC develop brain atrophy and secondary hydrocephalus (i.e., ex-vacuo dilation of the ventricles) and TBI and ischemic/hemorrhagic lesions will deform the brain considerably. On the one hand, these factors commonly restrict standardized voxel-wise ROI analysis (Hulkower et al., 2013) to a smaller and highly select sample of patient. On the other side, these factors have discouraged the use of finer but more difficult techniques, such as deterministic tractography, which requires an a priori positioning of selective ROIs for the reconstruction of specific WM fascicules in a distorted brain anatomy. Possible contamination with physiological artifacts (Chuang and Chen, 2001) (remaining unresolved using anesthetics), might represent another important issue regarding the study of DOC patients, although the use of triggered DTI sequences could attenuate these confounds. Finally, results regarding WM microstructural changes during follow-up exams may be conflicting due to the limited ability of DTI to resolve crossing and kissing fibers (Yeh et al., 2012). Advanced diffusion MRI techniques, such as HARDI or Spherical Deconvolution techniques, can help to resolve such conflicting results and provide an insight into neural connectivity that has not previously been possible *in vivo*. To summarize, the use of DTI in comatose patients has already increased our knowledge of DOC, particularly in the case of traumatic etiology, whilst the application of the DTI technique in the study of patients with AHI is all but absent. For now, the use of DTI to determine DOC severity, aid diagnosis and ascertain prognostic outcomes is limited to a research setting. Despite this, DTI has proved a powerful tool as it grants insight into the pathogenesis and specific WM abnormalities underlying different comatose states, casting light on the neural basis of consciousness and the clinical features associated with DOC.

### Conflict of interest statement

The authors declare that the research was conducted in the absence of any commercial or financial relationships that could be construed as a potential conflict of interest.
